# Salicylic acid had the potential to enhance tolerance in horticultural crops against abiotic stress

**DOI:** 10.3389/fpls.2023.1141918

**Published:** 2023-02-16

**Authors:** Shanshan Chen, Chun-Bo Zhao, Rui-Min Ren, Jun-Hai Jiang

**Affiliations:** College of Horticulture, Jilin Agricultural University, Changchun, China

**Keywords:** abiotic stress, osmotic stress, poor yield, plant metabolism, plant defense activities

## Abstract

Horticultural crops are greatly disturbed by severe abiotic stress conditions. This is considered one of the major threats to the healthy lives of the human population. Salicylic acid (SA) is famous as one of the multifunctional phytohormones that are widely found in plants. It is also an important bio-stimulator involved in the regulation of growth and the developmental stages of horticultural crops. The productivity of horticultural crops has been improved with the supplemental use of even small amounts of SA. It has good capability to reduce oxidative injuries that occur from the over-production of reactive oxygen species (ROS), potentially elevated photosynthesis, chlorophyll pigments, and stomatal regulation. Physiological and biochemical processes have revealed that SA enhances signaling molecules, enzymatic and non-enzymatic antioxidants, osmolytes, and secondary metabolites activities within the cell compartments of plants. Numerous genomic approaches have also explored that SA regulates transcriptions profiling, transcriptional apprehensions, genomic expression, and metabolism of stress-related genes. Many plant biologists have been working on SA and its functioning in plants; however, its involvement in the enhancement of tolerance against abiotic stress in horticultural crops is still unidentified and needs more attention. Therefore, the current review is focused on a detailed exploration of SA in physiological and biochemical processes in horticultural crops subjected to abiotic stress. The current information is comprehensive and aims to be more supportive of the development of higher-yielding germplasm against abiotic stress.

## Introduction

Agriculture production is decreasing because of the rapid human population growth and recent global climate change. It is necessary to improve and boost the required agricultural outputs by nearly 70% by the mid-century to feed huge populations in the future (Bulgari et al., 2019). Moreover, the production of horticultural crops is badly affected by climate change. Crop growth and yield is reducing gradually due to elevated environmental extremities occurring from industrialization and urbanization. Several biotic (e.g., insect pests and diseases) and abiotic stresses (e.g., drought, salinity, heavy metals, and temperature extremities) are major constraints restricting the productivity of horticultural crops (Devi et al., 2022). One of the most challenging aspects for plant researchers is plant response under diverse environmental stress conditions. Water deficiency, an excess of salts, and temperature extremities are major abiotic constraints disturbing plant health at growth, yield, and quality stages (Mangal et al., 2022). The development, characterization, and evaluation of tolerant germplasm is a recent need against abiotic stresses. However, while some traditional strategies have been adopted for the development of tolerant germplasm, they are laborious, time-consuming, and not capable of eliminating multiple stresses (Ahmad and Anjum, 2018; Ahmad et al., 2023). Therefore, appropriate measures are being developed to fulfill the food needs of the world’s population. Moreover, it is imperative to develop suitable and appropriate strategies in the current era to alleviate the problems of intolerance in horticultural crops (Mahpara et al., 2019). Hence, the exogenous application of phytohormones (including salicylic acid, abscisic acid, ascorbic acid, melatonin, brassinosteroids, strigolactones, jasmonates, auxins, ethylene, cytokinins, and gibberellins) may possibly be an accurate and concise option for the production of climate-resilient crops with higher yields and excellent quality.

Among phytohormones, SA has been considered an alternative eco-friendly and suitable chemical regulator for the alleviation of abiotic stress tolerance issues in many horticultural crops. SA is effective even in very small amounts in numerous ways for the regulation of plant growth and developmental stages in response to stressful conditions, as reported by Jiang and Asami (2018). Wani et al. found SA to be more effective for the regulation of different signal transduction trails in response to plants growing under abiotic stress (2016). Regulation of external and internal stimuli is encouraged due to SA, which further contributes to major changes occurring in the developmental processes of horticultural crops (Kazan, 2013). SA is widely known as one of the signaling molecules in horticultural crops subjected to abiotic stress (Jahan et al., 2019). Therefore, it has been assumed that SA is appropriate and effective for the mitigation of the adversities of abiotic stress. SA is also beneficial for the quality production of horticultural crops (Li et al., 2021). Numerous physiological, biochemical, and photosynthetic pigments and molecular mechanisms were regulated by the supplemental use of SA against abiotic stress. SA is also famous as one of the promising phytohormones with an excellent capability to cope with the adversities that occur from abiotic stress. SA has the potential to enhance the generation of bioactive compounds and activate plant defense systems against stressful conditions (Mahpara et al., 2019). Therefore, SA supplemental use is effective and supportive for the excellent production of horticultural crops growing under abiotic stress conditions.

The supplemental application of SA is necessary for the sustainable production of horticultural crops subjected to abiotic stress conditions, as it improves plant defense systems. Therefore, the exogenous use of phytohormones (SA) is the most effective and promising method for the alleviation of abiotic stress intolerance in horticultural crops. Thus, there is a need to explore the physiological and biochemical responses of plants with the exogenous use of SA against abiotic stress for sustainable yields.

## Impact of climate-related extremities on the growth and yield of horticultural crops

Horticultural crops are rich source of nutritional contents necessary for healthy life because of the higher nutritional value and capability of staple food for the biosphere. Horticultural crop production is disturbed globally due to abiotic stresses, i.e., water deficiencies, salinity, cold, heat, heavy metals, minerals deficiencies, UV light, and pesticides. Abiotic stresses are altering the morphological, physiological, anatomical, and biochemical processes in plants and have an adverse effect on production or plant senescence (Rao et al., 2016). It has been shown that plant response to stressful conditions is chiefly based on type, duration, level of stress, plant stage, and the genetic make-up of species/cultivars (Feller and Vaseva, 2014); this can result in restrictions in growth, poor and low yield, disturbances in developmental phases, the rupturing of photosynthetic pigments, membrane injuries, disruptions in photosynthesis and stomatal regulation, and a low water potential in leaves, as indicated by Wani et al. (2016).

Harsh climatic conditions are becoming more noticeable because of the rapid increase in pollution and extensive variations due to global climate change. For instance, water deficit conditions around the globe may possibly be raised due to persistent exposure to temperature extremities in rain-fed places. Higher temperature conditions in the future are likely to cause water shortages and excess salts in soils, as predicted by Raftery et al. (2017). Furthermore, they also revealed thattemperature is going to be increased due to global warming. However, production and quality decline in horticultural crops in the near future are likely to be due to multiple stresses like drought, salinity, cold, heat, mineral deficiencies, and heavy metals. Furthermore, it has been noted that 90% of agricultural regions are becoming more susceptible to single or multiple environmental stresses.

Transcriptional activities have a greater contribution to the tolerance to oxidative stress. Different researchers have revealed the significance of transcriptomic activities in the regulation of ABA production (Cao et al., 2014). Moreover, the ASC-GSH cycle is regulated at the maximum level in the tolerant-germplasm as compared to sensitive-germplasm, as reported by Mahajan and Sanejouand (2015). Moreover, it can also be used for the characterization of either tolerant or sensitive landraces for sustainable production (Gallardo et al., 2014).

Different management practices have been employed to cope with the adverse effects of abiotic stress in horticultural crops (Kazan, 2013). Agronomic practices, molecular approaches, and supplemental use of phytohormones are more appropriate practices for the alleviation of intolerance in horticultural crops against abiotic stress (Jiang and Asami, 2018; Jahan et al., 2019). Among phytohormones, SA is well known for its mitigation of adverse effects of abiotic stress. Previous plant researchers have revealed that SA has a good capability to regulate the uptake of minerals, stomata regulation, the excellent working of photosynthetic contents, the generation of metabolites, and the defense system by activating ROS scavengers against abiotic stress (Li et al., 2021). Therefore, appraising the associations of these environment-related extremities with plant morpho-physiological and biochemical responses is imperative for the improvement of numerous horticultural practices.

## SA is a safeguarding and signaling molecule for horticultural crops under abiotic stress

SA is a well-known molecule that can protect against abiotic stresses by acting as a signaling compound. Abiotic stress indication is necessary to cope with its adverse effects in a timely manner for higher yields with quality produce. Horticultural crops are rich in minerals and vitamins necessary for a healthy life. Therefore, management practices are imperative for higher yielding-germplasm of horticultural crops. Climate change and continuous cropping are threats to the productivity of horticultural crops through abiotic stress (Connor, 2002). Moreover, SA is not only important for the regulation and activation of the defense system of plants against biotic stress but also more effective and helpful for the improvement of abiotic stress tolerance in horticultural crops, as discussed by Horváth et al. (2007).

The fundamental appliances of SA-enhanced abiotic stress resistance comprise SA-mediated osmolytes generation and their accumulation. Osmolytes generation could be helpful for the maintenance of osmotic homeostasis, minerals and nutrients uptake and up-regulation, increasing the scavenging of ROS activities, and improving the productivity of secondary metabolites (such as glutathione, phenolics, phytoalexins, alkaloids, allicin, terpenes, thionins, defensins, and glucosinolates). SA signaling activates the osmolytes production necessary for the maintenance of numerous hormone pathways (Khan et al., 2015). Therefore, it has been assumed that SA is found to be an important signaling molecule, and also acts as a safeguard for the alleviation of intolerance in horticultural crops growing under climate-related extremities globally (**Tables 1**, **2**).

**Table 1:** Exogenous SA involvement against abiotic stress in vegetable production.

Stress type	Crop name	Impact	Reference
Chilling injury	Sponge gourd	1.5 mM L^−1^ found to be more effective for reduction of chilling injuries under storage	Cong et al. (2017)
Ultraviolet radiation	Pea	0.4 mM enhances growth by improving defense system	Martel and Qaderi (2016)
Heat stress	Potato	Nearly, 2, 3, 8, 14, and 21 dpi applied on Chicago and Gala cultivars resulting in improved physiological mechanisms	Makarova et al. (2018)
Chilling injury	Sweet potato	Chances of chilling injury were decreased with application of SA by improving antioxidant potential of plants	Hu-qing et al. (2014)
Chilling-induced oxidative damage	In eggplant	Oxidative injury was reduced with foliar application of SA of about 0.1 mM, with improved defense systems and over-expression of defense-related genes	Chen et al. (2011).
Chilling conditions	Potato	Production was improved by application of 0.1 mM to enhance chilling tolerance	Mora-Herrera et al. (2005).
Heat stress	Cucumber	1 mM exogenous spray improved the plant defense system through reduction of MDA, H_2_O_2_, and ROS; SA increased the photosynthetic pigments growing under temperature extremities	Shi et al. (2006)
Heavy metals	Melon	0.1 mM concentration necessary to decrease the adsorption of Cd levels, over-generation of ROS, enhanced oxidative enzymes, proline level, and protein content	Zhang et al. (2015)
Salinity	Garlic	Exogenous spray of SA 300 ppm revealed excellent vegetative and reproductive growth with enhanced level of minerals such as potassium and calcium, while a decrease in sodium and chloride contents was also recorded in the vegetable plants	Shama et al. (2016)
UV-B	Peppers	Approximately, 1.5 mM enhanced the antioxidant potential under stressful conditions	Mahdavian et al. (2008)
Salinity	cucumber	It showed noticeable decrease in Na^+^ with increased amounts of numerous minerals in plants	Yildirim et al. (2008).

**Table 2:** Exogenous SA involvement against abiotic stress in fruit production.

Stress type	Crop name	Key finding	Reference
Chilling tolerance	Banana seedlings	Approximately, 0.5 mM increased the capability of antioxidant activities. Decrease of oxidative stress indicating activities such as ROS, MDA, and H_2_O_2_	Kang et al. (2003).
Chilling tolerance	Peach	Stress tolerance in fruit crop *via* increase of different sugars, sugars metabolism, sucrose level, and activation of genes stress linked to cold responses	Zhao et al. (2021).
Heat stress	Grape leaves	A higher activation of rubisco activities was measured with the integration of the photosynthesis process. SA also improved the uptake of minerals content *via* roots toward other parts of the plant	Wang et al. (2010)
Salinity	Mango	Sukkary rootstock was found to be salt tolerant with application of SA at 200 mg L^−1^	Muhammed et al. (2022)
Malformation in mango	Mango	Two cvs. Amrapali and Dashehari were found to be more tolerant with application of 0.40% SA against fruitlet abscission and malformation *via* improvements in chlorophyll fluorescence, photosynthesis, and the defense system	Kumari et al. (2021)
Heavy metals	Mango	Keitt and Ewais cultivars were irrigated with sewage wastewater. Stunted growth was recoded in 11 year’s old fruit trees. However, SA improved the tree growth and quality of fruits of both cultivars by mitigation of adversities occurring from heavy metals	Helaly et al. (2018)
Heavy metals	Mulberry	Plants showed stunted growth due to irrigation with poor quality sewage wastewater	Gul et al. (2021)
Salinity	Olive	0.25 mM of SA improved plant growth growing under 100 mM NaCl. Hence, SA was effective for fruits against abiotic stress conditions with an improved defense system	Aliniaeifard et al. (2016)
Osmotic stress	Mulberry	Genomic endeavors and over-expression of genes were effective for regulation of abiotic stress conditions in plants	Checker and Khurana (2013)

Interestingly, SA has a large contribution to the expression of pathogens-related genes (*PR*) containing *PR1*, *PR2*, and *PR5*, as revealed by Ali et al. (2018). *PR* genes are fascinating due to their capability to reduce pathogen attacks. Furthermore, these genes are also involved in the reduction of abiotic stresses. Similarly, another study by Wu et al. (2016) also revealed that *PR* gene expression is also interesting and helpful for the mitigation of the negative effects of abiotic stress. Overexpression of transgenic tobacco *PR-1* is also effective for tolerance against heavy metals in peppers, as revealed by Sarowar et al. (2005). In another study by Hong and Hwang (2005), it has been shown that pepper *PR-1* overexpression is also involved in the improvement of tolerance in the *Arabidopsis* against salinity and drought stresses. However, more molecular approaches need to be investigated, and much is still necessary for the enhancement of tolerance against abiotic stress.

Plant microbes interaction with plant health is attracting more attention from plant researchers. However, research into the increased potential of microbes against abiotic stress is still limited and needs further attention from plant researchers. Some plant researchers have observed that SA is involved in the increase of SA levels in microbes, which is further necessary for plant health by increasing soil porosity and maximizing nutrient uptake chances in plants (Lundberg et al., 2012). Microbes enriched with endogenous SA levels had greater potential to increase plant tolerance against harsh climatic conditions either due to climate change or continuous cropping patterns (Lebeis et al., 2015).

## Involvement of salicylic acid in the reduction of oxidative injury

**Figure 1:**
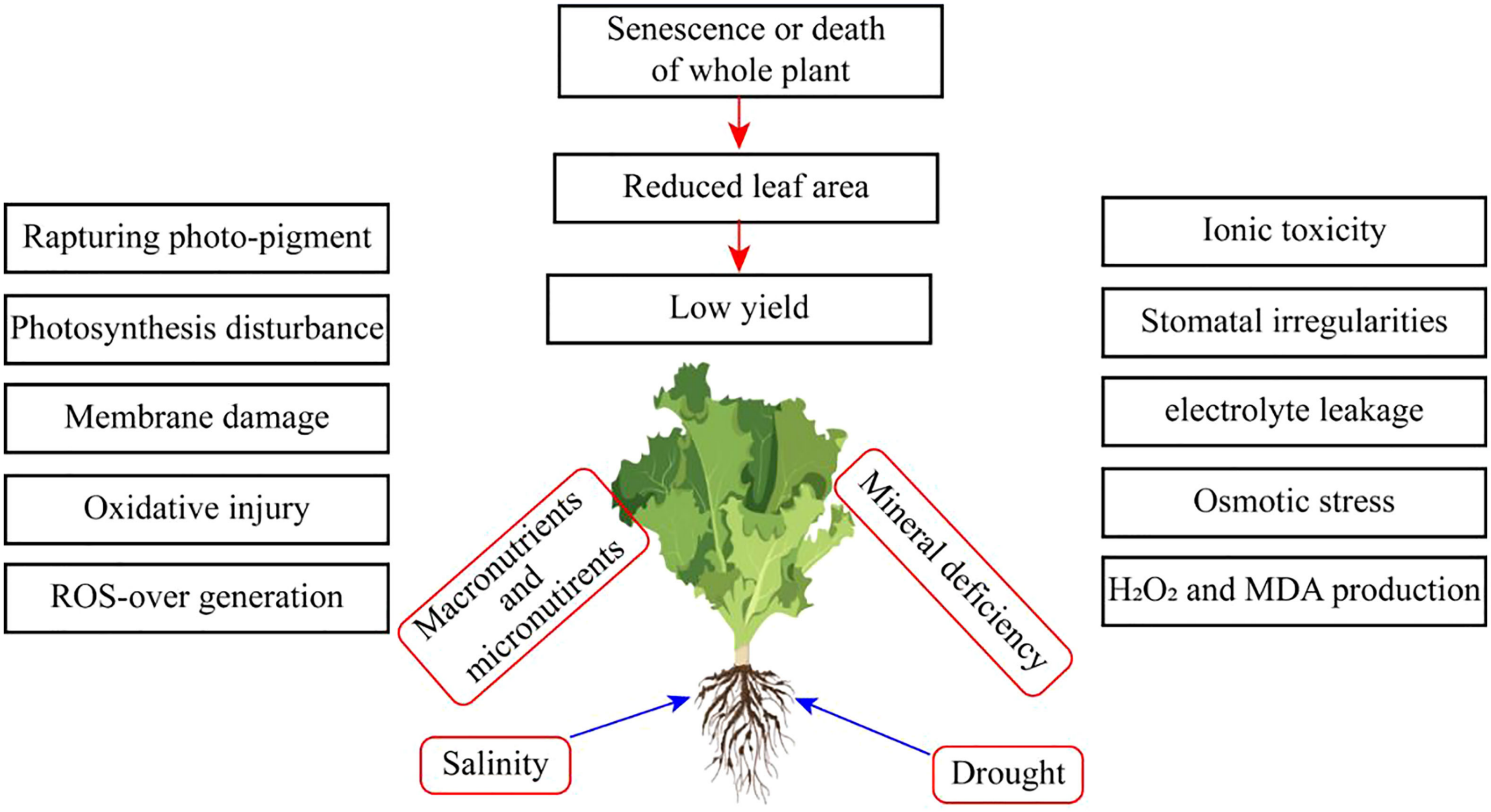
Effect of salinity and drought stress on horticultural crops.

**Figure 2:**
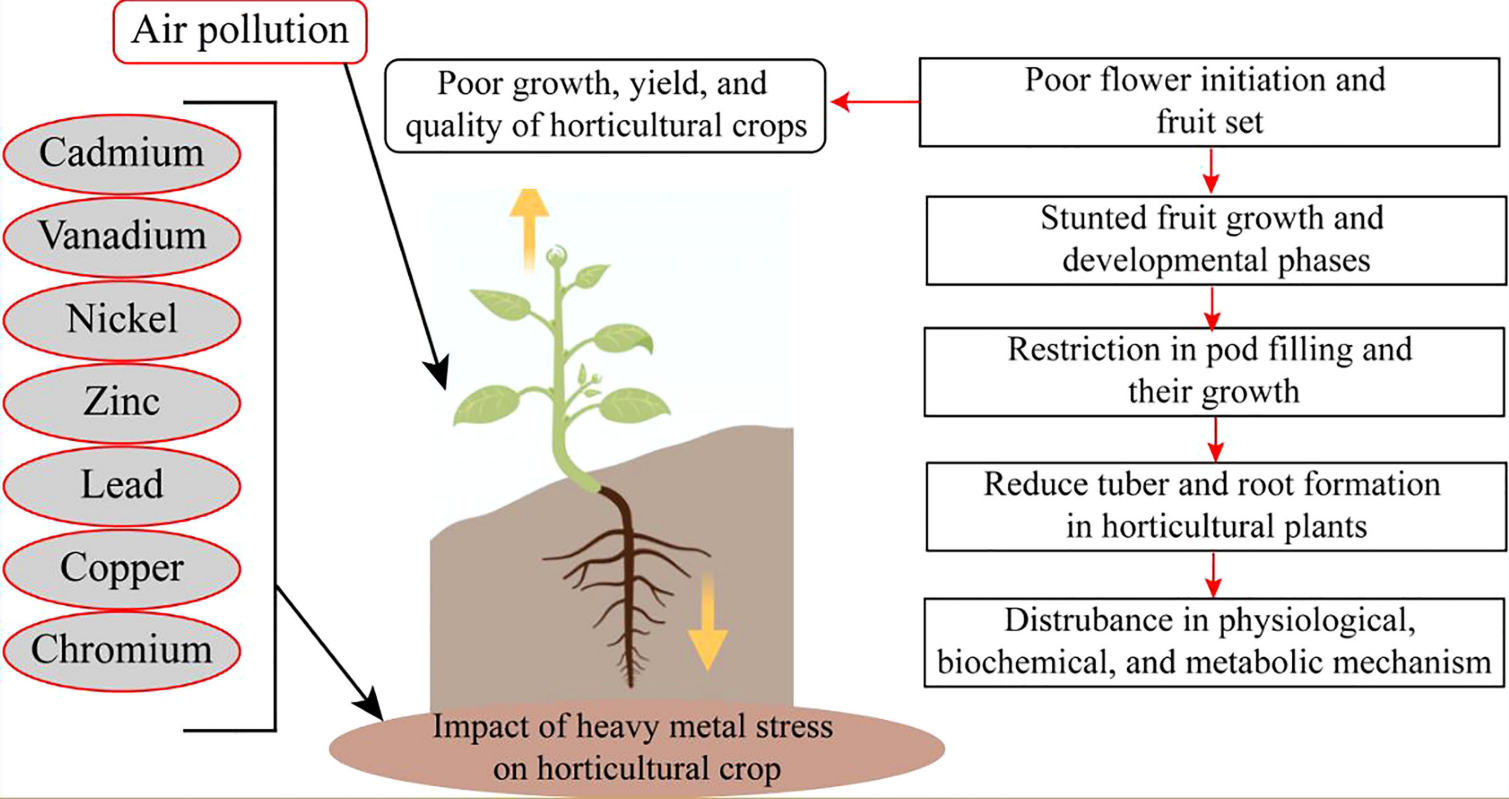
Heavy metals toxicity adversely affect the growth of horticultural crops.

Extremities of abiotic stress conditions result in excess toxic ROS. These potentially increase oxidative stress (**Figure 1**). However, ROS disturbs photosynthetic mechanisms, and the chance of osmotic stress occurs. Moreover, water stress conditions also majorly cause osmotic stress conditions in horticultural crops (**Figure 2**). Oxidative stress reduces plant growth, development, and yield. Cell membranes and electrolyte leakage are enhanced due to oxidative stress conditions. These conditions can damage parts or the whole of the plant (Muhammad et al., 2022).

Roots are considered as the first plant organ subjected to abiotic stress. Therefore, when exposed to abiotic stress, a higher reduction in growth and yield is observed in roots compared to other plant parts, like shoots. The elevated production of H_2_O_2_ reduces glutathione, and MDA indicates the extremities of oxidative stress conditions in the root zone, as studied by Ghosh et al. (2015). Elevated abiotic stress levels enhance the oxidative stress conditions in cells, compartments, and organelles. The increase of the accumulated photo-reducing effect is also due to oxidative stress. Furthermore, irregularities in the movement of electrons are mainly due to oxidative injuries in plants, as revealed by Sharma et al. (2012). Moreover, the Mehler reaction that occurs under abiotic stress deteriorates higher electrochemical energy and is considered one of the major causes of ROS, H_2_O_2_, and MDA activities at the extreme level.

The activation of enzymatic and non-enzymatic systems have the capability to trigger oxidative defense system under environmental stresses. However, it has been reported these responses to defense activities are chiefly organ specific. Polyphenols are extensively found in the root and young mature leaves. Therefore, these respond rapidly in roots and leaves compared to other plant parts. Polyphenol is famous as a non-enzymatic bioactive molecule involved in the defense system of plants (Tanou et al., 2009). However, the accumulation of tocopherols is not observed in roots because these are specific scavengers of singlet oxygen radicals in the photosystem II (Samira et al., 2015). In another study by Hussain et al. (2018), it was recorded that tocopherol accumulation was reduced in rice leaves due to excess salinity. Moreover, it has been found that plant roots have the potential to produce abscisic acid (ABA) under stressful conditions. ABA production is an indication of stress conditions in the root biosphere because it acts as a signaling molecule in plants under stress conditions.

**Figure 3:**
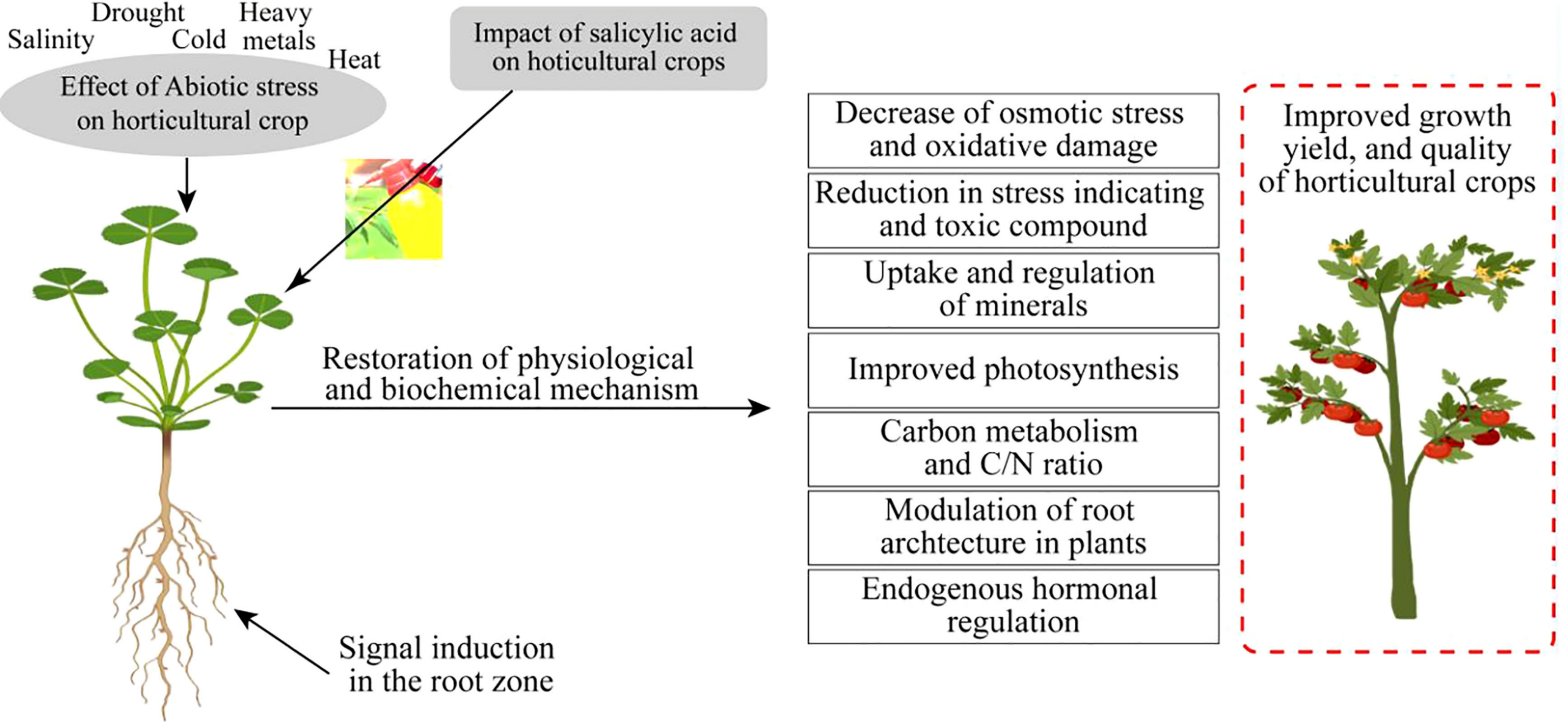
Impact of salicylic acid on horticultural crops under abiotic stress conditions.

Metabolic disturbances also occur due to oxidative extremities, as reported by Gallardo et al. (2014). Tolerant germplasm has the capability to produce an excess of tocopherols to cope with the adversities of stress conditions, as revealed by Sytykiewicz (2016). Furthermore, tocopherols have the potential to mitigate the negative effects of salinity even in seedling-stage plants. The exogenous application of SA is helpful to cope with the adversities that occur from abiotic stress. It is a multifunctional phytohormone with a diverse nature involved in the improvement of defense systems of numerous horticultural crops, i.e., bell pepper (Zhang et al., 2020), spinach (Gilani et al., 2020), peppermint (Ahmad et al., 2018), and potato (Li et al., 2019). Elevated growth, yield, photosynthesis, stomatal regulation, and protection from oxidative injury were due to the supplemental application of SA. Therefore, it can be assumed that SA is more effective for the alleviation of abiotic stress intolerance in horticultural crops focusing on higher yields with quality production (**Figure 3**).

## Salicylic acid improves salt tolerance by scavenging toxic ROS, MDA, and H_2_O_2_

SA is a multifaceted phenolic compound and naturally occurring molecule that signals against abiotic stress, as evaluated by Khan et al. (2015). SA contributes to the regular functioning of growth, photosynthetic adeptness, germination of seeds, roots enlargement, maturity and ripening, SA-microbes interaction, gravitropism maintenance, and responses to climate-related extremities, as described by Martel and Qaderi (2016). Supplemental use of about 1 mM of SA greatly increases water use efficiency (WUE), gas exchange mechanisms, activation of enzymatic activities, proline activation, and the reduction in oxidative injuries under extremities of temperature (heat stress) in tomatoes (Jahan et al., 2019). Biomass production is reduced due to drought stress. The disturbances in photosynthetic pigments and irregular stomatal functioning enhances the chances of osmotic stress. In another study, Galviz et al. (2021) demonstrated that 0.1 mM of SA improved biomass production, photosynthetic mechanism, enzymatic and non-enzymatic activities, and WUE, decreased cell-membrane damage, and provided better anatomical response under drought stress conditions. Similarly, Kaya (2021) revealed that SA had a better ability to induce drought tolerance in peppers by reducing oxidative injuries. Furthermore, photosynthetic behavior, enhanced proline levels, glyoxalase system, and regulation of stomata functioning were also reported with the utilization of SA in the peppers.

SA is effective for the mitigation of climatic adversities in temperate fruit crops by reducing oxidative and osmotic stress and improving defense systems. Chilling injury damage is most common in fruit crops grown in temperate regions. SA potentially provides tolerance to fruit crops through an increase in different sugars, sugars metabolism, sucrose levels, and the activation of genes related to cold responses, as described by Zhao et al. (2021). Chen et al. (2011) found that in eggplants growing under abiotic stress, oxidative injuries were reduced with a foliar spray of nearly 0.1 mM of SA (chilling injury), with improved defense systems and expression of defense-related genes. Potato production in chilling conditions was improved by the use of SA. Hence, 0.1 mM of SA was most effective to increase the chilling tolerance in plants, as demonstrated by Mora-Herrera et al. (2005). Similarly, another study by Shi et al. (2006) also evaluated that 1 mM of SA exogenously sprayed on cucumbers significantly improved the plant defense systems against heat stress through the reduction in MDA, H_2_O_2_, electrolyte leakage, and ROS. Moreover, SA improved the photosynthetic behavior of cucumbers against heat stress. Regarding heavy metals, Cd toxicity was reduced with 0.1 mM of SA in melons through the decreased adsorption of Cd, over-generation of ROS, and enhanced oxidative enzymes, proline levels, and protein content, as observed by Zhang et al. (2015). A better defense system indicated the tolerance level of a horticultural crop (potato) under stressful environments (heat stress) (Lopez-Delgado et al., 1998). Under heat stress, banana production improved with the exogenous use of SA, which improved the antioxidant pool, as examined by Kang et al. (2003). Furthermore, ROS, MDA, and H_2_O_2_ were reduced with SA application as a foliar spray. SA has the potential to mitigate adversities that occur due to abiotic stress by improving minerals uptake and ions homeostasis (Yalpani et al., 1994; Santisree et al., 2020).

The use of SA is an imperative way to lessen the adversities of abiotic stress and significantly enhance tolerance in horticultural crops of tropical and subtropical regions. A SA dose of 0.5 mM significantly enhances the activating potential of antioxidant activities in banana seedlings. However, the reduction in oxidative stress indicates activities such as ROS, MDA, and H_2_O_2_ (Kang et al., 2003). Moreover, higher activation of rubisco was recorded in grape leaves *via* the integration of photosynthesis under heat stress (Wang et al., 2010). SA improves mineral uptake from the roots toward other parts of the plant. Shama et al. (2016) demonstrated that an exogenous spray of SA 300 ppm revealed excellent vegetative and reproductive growth with enhanced level of minerals, such as potassium, while a decrease in sodium levels was recorded in garlic growing under salinity. SA is favorable for the growth of plants under normal or even stressful conditions. It produced a noticeable decrease in Na+ with increased amounts of numerous minerals in cucumbers against salinity stress, as described by Yildirim et al. (2008). A SA dose of 1.5 mM enhanced the antioxidant potential in peppers (Mahdavian et al., 2008), cucumbers (Lei et al., 2010), and peas (Martel and Qaderi, 2016) under UV-B stress.

## Salicylic acid and osmolytes activities

Abiotic stress may be detected by measuring signaling molecules generated within the plant’s body. Osmolytes are important signaling molecules for abiotic stress in horticultural crops, especially fruit crops (Rabey et al., 2016). Disturbances in the osmotic conditions in the root’s biosphere are due to higher salt levels and water deficiency. Osmoprotectants are also naturally produced for the activation of the plant defense system against stressful conditions. Over-production of toxic ROS and H_2_O_2_ were regulated by the generation of osmolytes like proline, glycine betaine (GB), and ascorbates, and their regulation was an indication of tolerance to abiotic stress, as reported by Munns (2002).

Elevated production of osmolytes showed a major involvement in the regulation of stomata conductance, transpiration, and respiration rates (Fu et al., 2019). Production of ascorbates within the plant cells could decrease the generation of H_2_O_2_ and numerous other derivatives, as evaluated by Fatemi et al. (2021). Moreover, stress intolerance can be alleviated by the production of osmolytes within the plant compartments. Osmolytes production is effective for the removal of the toxic effects of MDA content in plants under abiotic stress. Furthermore, ROS reduction indicators were also observed in plants due to the production of osmolytes (Racchi, 2013). Tolerant germplasm had less MDA and H_2_O_2_ generation compared to sensitive germplasm (Abbas et al., 2015). SA can be effective in the reduction of MDA and H_2_O_2_ activities.

Proline contributes to the stabilization of numerous protein structures and membrane protection against the adversities of ROS activity. These are excellent scavengers of over-generated ROS within the plant cells (Mittler et al., 2004). Elevated proline concentration has been revealed in tolerant germplasm; however, sensitive germplasm has a very poor generation of proline content within the cells and compartments. Therefore, proline concentration supports coping with the negative effects of abiotic stress. Numerous plant biologists have revealed that proline is a stress-signaling molecule (Rahneshan et al., 2018). Osmolytes GB were generated in maximum amounts for plants growing under climate-related extremities examined by Karimi and Kuhbanani (2015). Similarly, in another study by Abdollahi and Takhti (2013), the stability of photosynthetic pigments and protein structures was found to be regulated by the productivity of GB content. The characterization, evaluation, and cultivation of tolerant germplasm is vital for higher yields.

Phytohormones are important for increasing the antioxidant potential that promotes the defense systems of plants. These contribute to the scavengers of over-generated ROS and H_2_O_2_ (Haneklaus et al., 2007). Supplemental application of SA in plants also improved the antioxidant profiling and regulation of stomata conductance and photosynthesis, as observed by Molassiotis et al. (2016). Hence, SA is necessary for the improvement of defense processes of plants cultivated under abiotic stress for excellent growth and elevated yields. The exogenous use of SA on horticultural crops is still limited, and little information currently exists. Hence, the present review inspires the utilization of SA on horticultural crops for enhanced defense systems.

## Hormonal regulation with exogenous SA application

Endogenous hormonal regulation is necessary in cucumbers to increase tolerance against abiotic stress, as described by Sharif et al. (2022). The excellent potential of hormonal regulation was reported in two *Zizyphus* species when compared to others. The regulation of hormones could be involved in the maintenance of stomata conductance and photosynthetic activities, as reported in a study by Meena et al. (2003). Among supplemental phytohormones, SA is vital for enhancing resistance against stressful plant conditions. SA spray improves the antioxidant potential and also improves the scavenging potential for ROS, H_2_O_2_, and MDA, as reported by Mansour (2000). Similarly, Verma et al. (2018) reported that a reduction in stress indicators was due to hormonal regulation in fruit crops. Moreover, hormonal regulation and molecular programming are effective for abiotic stress tolerance in plants (Raza et al., 2022a; Raza et al., 2022b). Therefore, the use of SA and its exploration is important for the mitigation of adversities in stressful conditions focusing on crop yields. The application of SA at 100 mg L-1 has been evaluated by Rehman et al. (2011) to have significant and positive effects on the improvement of seed germination and the overall production of cucumbers growing in stressful conditions. The production of eggplants was increased with the improvement of endogenous hormonal levels by the application of 0.3% SA under stressful conditions, as evaluated by Chen et al. (2011).

## Conclusion and future concerns

The adversities of biotic and abiotic stresses are increasing due to the fluctuations in climate change in the present era. Different management practices can be used for the reduction of the negative impacts of abiotic stress on horticultural crops. Among these, supplemental use of phytohormones is effective for the sustainable production of horticultural crops against climate-related extremities. Among phytohormones, SA is more helpful and supportive in nature, and involved in the alleviation of stress intolerance in plants. It has been recommended that a small amount of SA has an excellent capability to cope with adversities that occur due to abiotic stress, and this is a good way to ensure the sustainable production of agricultural crops globally.

## Author contributions

SC and C-BZ: conceptualization, literature survey, writing original draft. SC and R-MR: writing – review and editing, figure designing. R-MR and J-HJ: figure designing, writing – review and editing. All authors contributed to the article and approved the submitted version.
